# Editorial: Phosphorylation-Dependent Peptidyl-Prolyl Cis/Trans Isomerase PIN1

**DOI:** 10.3389/fcell.2020.620418

**Published:** 2020-11-27

**Authors:** Jormay Lim, Tae Ho Lee, Futoshi Suizu

**Affiliations:** ^1^Department of Biomedical Engineering, College of Medicine and College of Engineering, National Taiwan University, Taipei, Taiwan; ^2^Fujian Key Laboratory for Translational Research in Cancer and Neurodegenerative Diseases, Institute for Translational Medicine, School of Basic Medical Sciences, Fujian Medical University, Fuzhou, China; ^3^Division of Cancer Biology, Institute for Genetic Medicine, Hokkaido University, Sapporo, Japan

**Keywords:** PIN1, peptidyl prolyl isomerization, cancer, Alzheimer's disease, phosphorylation

PIN1 has been known to isomerize the phosphorylated serine/threonine-proline (pS/pT-P) motif and exert its physiological function by regulating multiple phosphorylated signaling proteins via different mechanisms for one ultimate goal of the cell, for instance osteogenic differentiation, an example not covered by the articles in this Research Topic. In particular, PIN1 regulates osteogenesis via stabilizing RUNX2 or OSX from ubiquitination, or by increasing nuclear retention of β-catenin in Wnt3a-induced osteoblast (Yoon et al., 2013; Lee et al., 2015; Shin et al., 2016). Targeting the role of PIN1 in osteogenesis through pharmacological inhibition has been applied in the study of craniosynostosis due to FGFR2 mutation (S252W) in mice modeling Apert syndrome, suggesting that PIN1 is important for FGFR2 mutant (S252W)-induced RUNX2 activation (Shin et al., 2018). This is one of the most rewarding outcomes of the intensive research into PIN1 since the original discovery of its function in mitosis (Lu et al., 1996). With much accumulated and ever-expanding knowledge, we need up-to-date summaries organized into tables and charts to describe the ever-increasing roles of PIN1 and its binding partners, so that more studies are inspired.

The idea that PIN1 could be used as a molecular switch and the concept of PIN1 catalyzing by lowering the energy barrier for cis- and trans- isomerization for pS/pT-P, accelerating the conversion up to a 1,000-fold, has been previously proposed (Liou et al., 2011). In this Research Topic, Chen et al. summarizes the understanding of how PIN1 is regulated, with a comprehensive table on the post-translational modifications (PTMs) of PIN1, including phosphorylation, oxidation, SUMOylation, and ubiquitination. How PTMs regulate PIN1 enzymatic activity, binding ability, localization, and function, and how the deregulation of PIN1 PTMs contribute to the development of cancer and Alzheimer's disease (AD), are reiterated and conceptualized. Therapeutic options and the challenge for targeting PIN1 PTMs with possible drug candidates are also discussed.

PIN1 promotes cell growth and proliferation, and its aberrant expression and activity are associated with cancer development. The mini review written by Pu et al. highlights PIN1's biological mechanisms, listing the deregulatory factors of PIN1 at transcriptional, translational, and post-translational levels. PIN1's roles manifest in its substrates and their biological activity, protein stability, and cellular localization in different types of cancer. The authors discuss the multifaceted roles of PIN1 in proliferative signaling, growth, invasion, metastasis, and angiogenesis during oncogenesis, with an impressive list of the substrates' specific phosphorylation sites. Cheng and Tse's review emphasizes the importance of PIN1 in regulating hepatocellular carcinoma (HCC). PIN1 meddles in HCC development by impairing microRNA biogenesis, enhancing cell proliferation, inhibiting apoptosis, and promoting migration, molecularly involving and interacting with cyclin D1, Hepatitis B virus X-protein, exportin-5, and GLI1. The development of PIN1 inhibitors, such as sorafenib, all-trans retinoic acid (ATRA), arsenic trioxide, and API-1, for HCC treatments are organized in a table. Yu et al. focuses on recent cancer drug development targeting PIN1 in a variety of human malignancies, especially small-molecule compounds. They meticulously list more than 20 PIN1 inhibitors developed by PPIase relevant binding assays, structure-based rational design, and mechanism-based high-throughput screens, revealing the properties of recently discovered PIN1 inhibitors, working mechanisms, kinetics, and limitations in different cancer types.

In addition to the reviews, Huang et al., show in their original research that the treatment of the PIN1 inhibitor, ATRA, reduces PIN1 levels, thereby promoting ERα degradation and decreasing ERK1/2 and AKT activity in tamoxifen-resistant human breast cancer cells. These results suggest that ATRA inhibits multiple PIN1-drived cancer promoting pathways and provide a potential therapeutic strategy for treating drug-resistant cancers.

However, PIN1 sometimes acts in a double-edged sword manner. Makinwa, Musich et al. further elaborate on the complexity of PIN1 in cancer development and provide an anti-tumor role of PIN1 under certain conditions. Nevertheless, they focus on ataxia telangiectasia- and Rad3-related (ATR), recently discovered as a novel PIN1 substrate that plays an important role in DNA damage response. They discuss the PIN1-mediated *cis* to *trans* induced conformational change of ATR that promotes its anti-apoptotic function in certain types of cancer cells. Makinwa, Cartwright et al. have also found that protein phosphatase 2A (PP2A) dephosphorylates the PIN1 binding site of ATR, thereby accumulating the anti-apoptotic *cis* ATR in the cytoplasm. These data describe that PP2A may regulate PIN1 by depleting phosphorylated ATR, eventually causing cell death upon DNA damage.

PIN1 regulates a plethora of transcription factors and transcription cofactors including c-Myc, p53, and b-catenin. These substrates are organized into a table by Hu and Chen. The gene transcription governed by PIN1 substrates contributes to the diverse pathophysiological functions of PIN1, including cancer, neurodegenerative disorders, inflammation, and immune response. Understanding the PIN1-related transcriptional regulation of cell cycles provides new insights into the pathophysiological function of PIN1, and new strategies of therapeutic treatment for several PIN1 disfunction-related diseases. Cohn et al. provides a new perspective and they found that PIN1 interferes with the spatiotemporal dynamics of Myc via stabilizing its pS62. Of special interest is that this association facilitates the localization of phosphorylated Myc in the inner basket of the nuclear pore, thereby affecting the euchromatin, implying an epigenetic regulation.

PIN1 expression and activity are significantly suppressed in the brains of people with Alzheimer's disease (AD). Wang et al., addresses a major impact of PIN1 deregulation in AD development and the many ways in which PIN1 acts on based on currently recognized molecular mechanisms. They also discuss the developing diagnostic and therapeutic strategies targeting PIN1 and its upstream regulators.

In the dynamic interplay between pathogens, such as viruses or parasites, and the host in infectious diseases, host PIN1 can bind in an inter-species manner to the virus core protein. Nishi et al., discover that hepatitis B virus core protein (HBc) is a unique substrate of host PIN1. HBc is stabilized by PIN1 in a phosphorylation-dependent manner. They also show that the pyruvate dehydrogenase phosphatase catalytic subunit 2 (PDP2) can dephosphorylate HBc at the PIN1-binding sites, thereby suppressing PIN1-mediated HBc stabilization, implicating the possibility of designing new antiviral therapeutics based on targeting PIN1.

On the other hand, the parasite-derived PIN1 protein can isomerize host proteins, such as transcription factors. Medjkane and Weitzman discuss the function of parasitic protists *Theileria annulata* secreted PIN1 (TaPIN1), which regulates the activity of host transcription factors c-Jun and HIP1a by manipulating the ubiquitination of host interactor proteins Fbw7 and PKM2. *Theileria* hijacks the host transcriptional pathways to boost host cell proliferation and metabolic activity and maintains their critical nutrients for parasitic proliferation and survival within host cells. Therefore, TaPIN1 can be a critical target for treating infectious disease.

While the Research Topic collections summarize important recent findings of PIN1-mediated biological mechanisms and attempt to organize the vast amount of information relevant to the PIN1 interactions with its substrates, we have to admit that this series does not cover all the PIN1 research fields; as an example, PIN1 regulations of lipid and glucose metabolism (Nakatsu et al., 2020) is not discussed. We hope this Research Topic attracts attention to and questions on the further investigation of PIN1 biology.

## Author Contributions

JL coordinates, integrates, re-organizes, and finalizes the content of editorials. THL summarizes the manuscripts relevant to cancer and Alzheimer's diseases. FS summarizes the manuscripts relevant to pathogen-host interspecies PIN1 and substrate interactions and transcriptional factors and regulations. All authors contributed to the article and approved the submitted version.

## Conflict of Interest

The authors declare that the research was conducted in the absence of any commercial or financial relationships that could be construed as a potential conflict of interest.

## References

[B1] Lee S. H. Jeong H. M. Han Y. Cheong H. Kang B. Y. Lee K. Y. (2015). Prolyl isomerase Pin1 regulates the osteogenic activity of Osterix. Mol. Cell. Endocrinol. 400, 32–40. 10.1016/j.mce.2014.11.017 25463757

[B2] Liou Y. C. Zhou X. Z. Lu K. P. (2011). Prolyl isomerase Pin1 as a molecular switch to determine the fate of phosphoproteins. Trends Biochem. Sci. 36, 501–514. 10.1016/j.tibs.2011.07.001 21852138 PMC3185210

[B3] Lu K. P. Hanes S. D. Hunter T. (1996). A human peptidyl-prolyl isomerase essential for regulation of mitosis. Nature 380, 544–547. 10.1038/380544a0 8606777

[B4] Nakatsu Y. Yamamotoya T. Ueda K. Ono H. Inoue M. K. Matsunaga Y.. (2020). Prolyl isomerase Pin1 in metabolic reprogramming of cancer cells. Cancer Lett. 470, 106–114. 10.1016/j.canlet.2019.10.043 31678165

[B5] Shin H. R. Bae H. S. Kim B. S. Yoon H. I. Cho Y. D. Kim W. J.. (2018). PIN1 is a new therapeutic target of craniosynostosis. Hum. Mol. Genet. 27, 3827–3839. 10.1093/hmg/ddy252 30007339 PMC6216213

[B6] Shin H. R. Islam R. Yoon W. J. Lee T. Cho Y. D. Bae H. S.. (2016). Pin1-mediated modification prolongs the nuclear retention of beta-catenin in Wnt3a-induced osteoblast differentiation. J. Biol. Chem. 291, 5555–5565. 10.1074/jbc.M115.698563 26740630 PMC4786698

[B7] Yoon W. J. Islam R. Cho Y. D. Woo K. M. Baek J. H. Uchida T.. (2013). Pin1-mediated Runx2 modification is critical for skeletal development. J. Cell. Physiol. 228, 2377–2385. 10.1002/jcp.24403 23702614 PMC4051422

